# Storage and release of rare earth elements in microsphere-based scaffolds for enhancing osteogenesis

**DOI:** 10.1038/s41598-022-10347-0

**Published:** 2022-04-16

**Authors:** Weikang Xu, Kun Wei, Zefeng Lin, Tingting Wu, Guixiang Li, Liyan Wang

**Affiliations:** 1 Department of Scientific Research, Institute of Biological and Medical Engineering, Guangdong Academy of Sciences, National Engineering Research Center for Healthcare Devices, Guangdong Key Lab of Medical Electronic Instruments and Polymer Material Products, Jianghai Avenue Central (Former No.10, Pomegranate Gang Road), Haizhu District, Guangzhou, 510316 Guangdong China; 2 Department of Stomatology, Foshan Woman and Children’s Hospital, No. 11 Renmin Xi Road, Chancheng District, Foshan, 528000 Guangdong China; 3 National Engineering Research Center for Human Tissue Restoration and Function Reconstruction, South China University of Technology, Wushan Road 381, Guangzhou, 510006 Guangdong China; 4 Guangdong Key Lab of Orthopedic Technology and Implant Materials, General Hospital of Southern Theater Command of PLA, Guangzhou, 510010 Guangdong China

**Keywords:** Chemistry, Materials science

## Abstract

In osteoporosis and diabetes, it is essential to accelerate the bone repair and regeneration process. Trace rare earth elements such as lanthanum (La) ions (La^3+^) with appropriate concentrations are bioactive and can effectively regulate bone tissue performances. However, few well-established bone tissue engineering scaffolds can precisely and stably release La^3+^ to promote bone regeneration significantly. Based on the advantages of biodegradable microspheres and microsphere-based scaffolds for controlled drug release, we developed poly(lactide-co-glycolide) (PLGA)-based microsphere-based scaffolds as both three-dimensional (3D) porous scaffolds and La^3+^ storage and release systems for osteogenesis. So far, there is no study about microsphere-based scaffolds to release trace La^3+^ to induce osteogenic differentiation of bone marrow mesenchymal stromal cells (BMSCs). PLGA microspheres co-embedded with La-doped mesoporous silica (LMS) with different amounts of doped La were sintered to prepare the LMS/PLGA (LMSP) microsphere-based scaffold. The La^3+^ release behavior of LMSP can be controlled by adjusting the doping amount of La in mesoporous silica (MS). All these scaffolds possessed a 3D network architecture. With the increase of La doping, LMSP can better compensate for the pH decrease caused by PLGA degradation. The combination of MS and PLGA can avoid the cytotoxicity of MS alone. All prepared LMSP scaffolds were non-cytotoxic. After BMSCs were implanted on scaffolds, LMSP could promote cells adhesion, proliferation, and osteogenic differentiation. Among these microsphere-based scaffolds, LMSP-3 with stable and higher dose La^3+^ release behavior showed the strongest ability to enhance the osteogenesis of BMSCs. The results showed that microsphere-based scaffolds with the ability to store and stably control the release of La^3+^ could effectively improve osteogenic performance, which provides a new idea for the construction of bone tissue engineering scaffolds.

## Introduction

The treatment of bone defects caused by trauma, tumors, or infection remains a major challenge^1^. The autograft or allograft was used in clinical now^2^. Due to the availability, donor-position morbidity, additional pain, and infection risk, the clinical application and popularization of these two types of bone grafts are limited^3^. Based on tissue engineering, bone repair material assembled from scaffolds, cells, and growth factors has been developed and is an effective mode for successful bone repair^4^. Scaffolds serve as carrier materials for cell adhesion, migration, proliferation, and differentiation, play a significant role in the regeneration and repair of bone tissues^5^. With a view of the chemical compositions of natural bone, a variety of bioceramics have been incorporated into scaffolds. Among these inorganic components, bioactive elements including strontium, magnesium, copper, and zinc are widely investigated, in addition to hydroxyapatite (HA), calcium phosphate bone cement, bioactive glass, and mesoporous silica (MS)^6–8^. These trace elements can regulate the formation of new bone, which is beneficial to bone regeneration and repair^9^.

Lanthanum (La) is considered a "bone-seeking" element due to some physicochemical characteristics similar to calcium^10–12^. The accumulation of lanthanum ions (La^3+^) in the bone may enter the crystal lattice or deposit on the crystal face of HA, thus affecting bone minerals. La-based compounds or complexes have been used as activators in the treatments of bone density disorders, which stimulate bone formation without accelerating bone resorption^13^. However, the biological properties of rare earth elements, including La, are thought to be concentration-dependent effects. Low concentrations of La show beneficial and positive effects, while high concentrations of La are harmful to healthy cells/tissues^14^. For example, low concentrations of LaCl_3_ (10^–9^ M) enhanced the proliferation of bone marrow mesenchymal stromal cells (BMSCs). However, when the concentration was maintained at 10^–5^ M, LaCl_3_ induced cell apoptosis^15^. For La^3+^ ions to exert positive effects and avoid toxic effects on organisms and cells, they must be controlled within a safe concentration range. Studies have shown that La-doped inorganic ceramics could stimulate and enhance osteogenic differentiation. Especially the La-doped bioceramics or their composites with polymer biomaterials^16,17^. However, due to the degradation performance of bioceramics, it is difficult to control the release of trace La^3+^ in doped bioceramics^18^.

As a ubiquitous environmental element, silicon (Si) also plays a significant role in regulating the metabolic process of connective tissue, including bone^19^. The inorganic ceramic MS is a typical silicon-based material with good physical properties^20^. In the preparation process of MS, high-temperature calcination can be used to reduce the degradation rate of MS, making MS a good carrier material for the release of trace La^3+^^21^. However, MS particles with specific shapes and sizes make them unsuitable for bone repair alone. A polymer such as poly(lactic acid glycolide) (PLGA) is a variety of synthetic and biodegradable polymer that is used widely for bone tissue engineering scaffolds. It has good biocompatibility and physical and mechanical properties, such as adjustable degradation characteristics and processing capacity, can prepare the flexible structure, and has a customized degradation rate^5,22^. However, PLGA has poor adhesion to cells, lack of active functional groups, poor mechanical strength, degrades to water and CO_2_ within the body, and access CO_2_ leads to increased acidity in the vicinity, resulting in limited application in the biomedical field. It is a direct and plain modification method by hybridizing PLGA with MS that could effectively improve the compressive strength of the polymer scaffold^23^.

Because of their incomparable advantages in controlled release, microspheres are often used as drug and bioactive ion delivery materials^24–26^. The shape of the microspheres is rigid and can be used alone or in combination with other biomaterials to form tissue-engineered scaffolds with three-dimensional (3D) porous structures. The technology of using microspheres alone to prepare scaffolds has attracted wide attention, and the bottom-up preparation mode is becoming more and more popular^27^. Sintered microsphere technology is an effective method for preparing sintered microsphere-based scaffolds. It agglomerates microspheres together by heat or solvent^28^. Bone tissue engineering scaffolds must have 3D porous structure and good mechanical properties, and sintered microsphere-based scaffolds have these characteristics^23^. Because the microspheres in the scaffolds stick to each other, the scaffolds implantation can prevent the microspheres from escaping from the defect site. In addition, arthroscopic delivery devices can be used to achieve minimally invasive treatment^29^. Some studies have confirmed that sintered microsphere-based scaffolds have good biocompatibility and tissue repair properties^23,30–32^. So far, no literature has reported that PLGA-based microspheres or microsphere-based scaffolds as the release carrier of trace La^3+^ have been used to induce osteogenic differentiation of BMSCs.

In this study, microspheres were prepared using the well-known biodegradable PLGA to encapsulate La-doped mesoporous silica (LMS) with different amounts of doped La via a solid-in-oil-in-water (S/O/W) emulsion technique^33^. And the microsphere-based scaffolds were prepared with sintered microsphere technique. As far as we know, LMS/PLGA (LMSP) microsphere scaffolds for bone tissue engineering are rarely reported so far. The La^3+^ release behavior of scaffolds and its correlation with Alkaline phosphatase (ALP) secretion and osteogenic differentiation of BMSCs induced by scaffolds were investigated. At the same time, the change of pH of the medium was studied during the scaffold degradation. This work will lay a foundation for designing excellent bone tissue engineering scaffolds.

## Materials and methods

### Materials

Ethyl alcohol (EtOH), Tetraethoxysilane (TEOS), and dichloromethane (DCM) were purchased from Chemical Reagent Factory (Guangzhou, China). Lanthanum nitrate (La(NO_3_)_3_·6H_2_O) was purchased from Aladdin Chemistry Co. Ltd (Shanghai, China). PLGA (Mw = 31,000 g mol^−1^) with a ratio of lactic to glycolic acid of 50:50 was purchased from Daigang Biomaterials (Jinan, China). Polyvinyl alcohol (PVA) and dodecylamine (DDA) were obtained from Sigma–Aldrich (Singapore). Reagents for cell culture were purchased from Gibco (Carlsbad, Ca, USA). CCK-8 was produced in Dojindo, (Kumamoto, Japan).

### Preparation of LMS and LMSP microspheres scaffolds

Traditional techniques were used to prepare MS^23^. LMS was prepared as follows: DDA was dissolved in EtOH/deionized aqueous solution (pH = 9). Then, 0.5 g, 1.0 g, 1.5 g, or 2.0 g of La(NO_3_)_3_·6H_2_O and TEOS were added as sources of La and Si, respectively, and stirred. The molar composition of the reaction mixture is TEOS: 1.0, DDA: 0.27, EtOH: 9.12, H_2_O: 29.6. The mixture was stirred for 18 h, aged at room temperature for 12 h, dried at 90 °C for 6 h, cleaned with ethanol, and calcined to remove DDA. The LMS prepared by adding 0.5 g, 1.0 g, 1.5 g, and 2.0 g La source were labeled separately as LMS-1, LMS-2, LMS-3, and LMS-4.

MSP and LMSP microspheres were all prepared by the following two methods: the single-emulsion solvent volatilization method and the S/O/W method. In short, 1 g PLGA and 0.15 g MS or La-MS particles were dissolved in 5 mL DCM and mixed within 2 min. The synthetic mixture was then injected into a 1.0% PVA solution and stirred for 10 h at 300 rpm to evaporate the solvent completely. Microspheres were separated and washed with deionized water 5 times. MSP and LMSP microspheres were poured into a cylindrical mold and sintered at 70 °C for 2 h. The products of MS, LMS-1, LMS-2, LMS-3, and LMS-4 added LMSP microsphere-based scaffolds were labeled as MSP, LMSP-1, LMSP-2, LMSP-3, and LMSP-4, respectively.

### SEM analysis

The morphology of scaffolds was characterized by scanning electron microscopy (SEM, 30XLFEG, Philips, The Netherlands). The elemental compositions of the MS powder and microsphere-based scaffolds were analyzed using an energy dispersive spectrometer (EDS) in conjunction with the SEM system.

### Density and porosity determination

The density and porosity of sintered microsphere-based scaffolds were tested refer to Ref.^34^. Briefly, EtOH is selected as the liquid phase, and the temperature is kept at 25 °C. The bottle is filled with EtOH (weight W_1_), the cylindrical scaffolds (diameter 10 mm, height 20 mm, weight W_S_) is put into the bottle, and an equal volume of ethanol spills out. The bottle filled with the scaffolds and the remaining ethanol weighs W_2_. ρ is the density of EtOH at 25 °C. Using the following formula to calculate the porosity (P) and density (D) of the scaffold:

1 $$\mathrm{D}={\mathrm{W}}_{\mathrm{S}}/\uppi \times {\mathrm{R}}^{2}\times \mathrm{H},$$

2 $$\mathrm{P}=1-\left({\mathrm{W}}_{1}-{\mathrm{W}}_{2}+{\mathrm{W}}_{\mathrm{S}}/\uppi \times {\mathrm{R}}^{2}\times \mathrm{H}\times\uprho \right).$$

### Static contact angles

150 mg MS and LMS-1–4 particles were dispersed in DCM containing 1 g PLGA and poured into a glass dish. After the DCM was volatilized completely, MS/PLGA and LMS-1–4 /PLGA membranes were obtained. Static contact angles were detected by a contact angle analyzer (First Ten Ǻvngstroms, Virginia, USA) using the sessile drop technique at room temperature. Deionized water droplets of 50 µL are deposited on the film's surface at a rate of 5 μL/s through a gauge dispensing needle.

### Compressive testing

Cylindrical scaffolds with 10 mm in length and 5 mm in diameter were used for compressive testing through an Instron mechanical testing machine (Instron model 5544, Canton, MA). The crosshead speed is 5 mm/min at ambient temperature and humidity. Determine the maximum compressive strength of the scaffolds by using Merlin software.

### Release behavior of La^3+^ and change of pH value

To evaluate the release of La^3+^, 100 mg microsphere-based scaffolds were soaked in 30 mL PBS at 37 °C. On days 3, 7, 14, 21, and 28, 10 mL of sample solution was taken out and then analyzed by inductively coupled plasma optical emission spectrometry (ICP, PerkinElmer, Optima 7000DV, USA). To continue releasing, add fresh 10 mL PBS.

The cylindrical sintered microsphere scaffolds with a diameter of 10 mm were soaked in 10 mL PBS (pH = 7.4) at 37 °C. An acidity meter (Schott Instruments, Germany) was applied to determine the pH of the PBS.

### Cell culture on LMS and microsphere-based scaffolds

BMSCs were purchased from American type cultured specimens (ATCC, Manassas, VA). For MS and LMS-1–4 powders, the cells were resuspended in a fresh culture medium and then seeded at the density of 2 × 10^3^ cell/well on MS or LMS-1–4 for studies on cell proliferation. The concentration of MS and LMS-1–4 in every well was 0 ppm, 50 ppm, 100 ppm, 200 ppm, and 400 ppm, respectively. For studies on cell proliferation on microsphere-based scaffolds, cells were seeded on the scaffolds (diameter = 10 mm, height = 5 mm) at a density of 1 × 10^5^ cells/scaffold, and then DMEM containing 10% fetal bovine serum (FBS) was added for culture.

### Cell proliferation

The cell proliferation of each scaffold was analyzed quantitatively by the CCK-8 method. In short, the medium was removed and the cells were washed twice with PBS (pH = 7.2) at specified intervals. CCK-8 solution was added to each well and incubated in an incubator for 2 h. The absorbance was measured at 450 nm wavelength with the microplate reader (Thermo3001, America). Twenty-four hours after the cells were inoculated with the scaffolds, live/dead assay was performed using the calcein-AM/propidium iodide double staining kit (Sigma).

### Osteogenic differentiation

The osteogenic differentiation of BMSCs was observed by culturing the cell scaffolds (2 × 10^5^ cells/scaffold) with osteogenic medium (OGM). ALP activity was quantified using the p-nitrophenyl phosphate liquid substrate (Sigma Diagnostics) method. In short, the cell scaffolds were prewashed with PBS, and then the adherent cells were removed from the scaffold and lysed at 4 °C for 10 min, then dissolved in 0.5 mL PBS containing 0.1 M glycine, 1 mM MgCl_2_, and 0.05%Triton X-100 for 10 min. Then it was incubated with p-nitrophenyl phosphate (pNPP) solution at 37 °C for 30 min. To extract the cell layers, the cells were treated with sonication twice for 30 s and then centrifuged at 12,300 rpm for 2 min at 4 °C. ALP activity was assayed in the supernatant, as in Ref.^35^. The ALP values (U/µg) were normalized to protein content using the Micro BCA Protein Assay Kit (Pierce). Meanwhile, ALP activity was detected by ALP staining on day 10. In brief, the cell scaffolds were washed with PBS and fixed with 10% neutral formalin solution for 30 min. The cell scaffolds were dyed with BCIP/NBT at 37 °C for 30 min and then washed with distilled water.

On day 21, the collagen secretion of cells on the scaffolds was detected by Sirius Red staining^35^. After PBS washing and fixation, 0.1% Sirius red (Sigma) staining showed collagen. Unbound stains were washed with 0.1 M acetic acid before taking photos. Collagen type I (COL-I) were detected using the COL-I enzyme-linked immunosorbent assay (ELISA) kit (Thermo Fisher).

The RNAs of BMSCs were extracted after being cultured for 21 days for reverse transcription real-time polymerase chain reaction (qRT-PCR) evaluation. Four genes related to osteogenesis were selected for analysis. That is bone morphogenetic protein 2 (BMP2), osteocalcin (OCN), COL-I, and RunT-associated transcription factor 2 (RUNX-2). The primers used in this study were designed by Primer Premier 6 software (Premier Biosoft, USA) and are listed in Table 1. Cell/scaffold complexes were crushed in a frozen state using liquid nitrogen, and then the Trizol RNA extract kit (Invitrogen, Thermo Fisher) was used to extract the total RNA. According to the primers shown in Table 1, the qTOWER RT-PCR system (Toyobo, Japan) was applied to perform analysis. And data was processed by the ΔΔCt method with 18S-rRNA as the normalization standard.

**Table 1:** Primer sequences of selected genes to osteogenic differentiation of BMSCs.

Gene	Forward primer (5′–3′)	Reverse primer (5′–3′)
BMP-2	GGAAAACTTCCCGACGCTTCT	CCTGCATTTGTTCCCGAAAA
OCN	CAAACACGGCAAGGTGTGTGA	CGAAGGTCTTGTTGTCATTGCTG
COL-I	GCGGTGGTTACGACTTTGGTT	AGTGAGGAGGGTCTCAATCTG
Runx-2	CCCAAGCATTTCATCCCTCACT	CATACCGAGGGACATGCCTGA
18S	GTAACCCGTTGAACCCCATT	CCATCCAATCGGTAGTAGCG

### Statistical analysis

The experiments were repeated five times, and the results were expressed as mean ± standard deviation. The results were calculated by one-way ANOVA and were statistically significant. Tukey test was used for mean comparison, and the difference was statistically significant as p < 0.05.

## Results

### Physical properties of scaffolds

**Figure 1:**
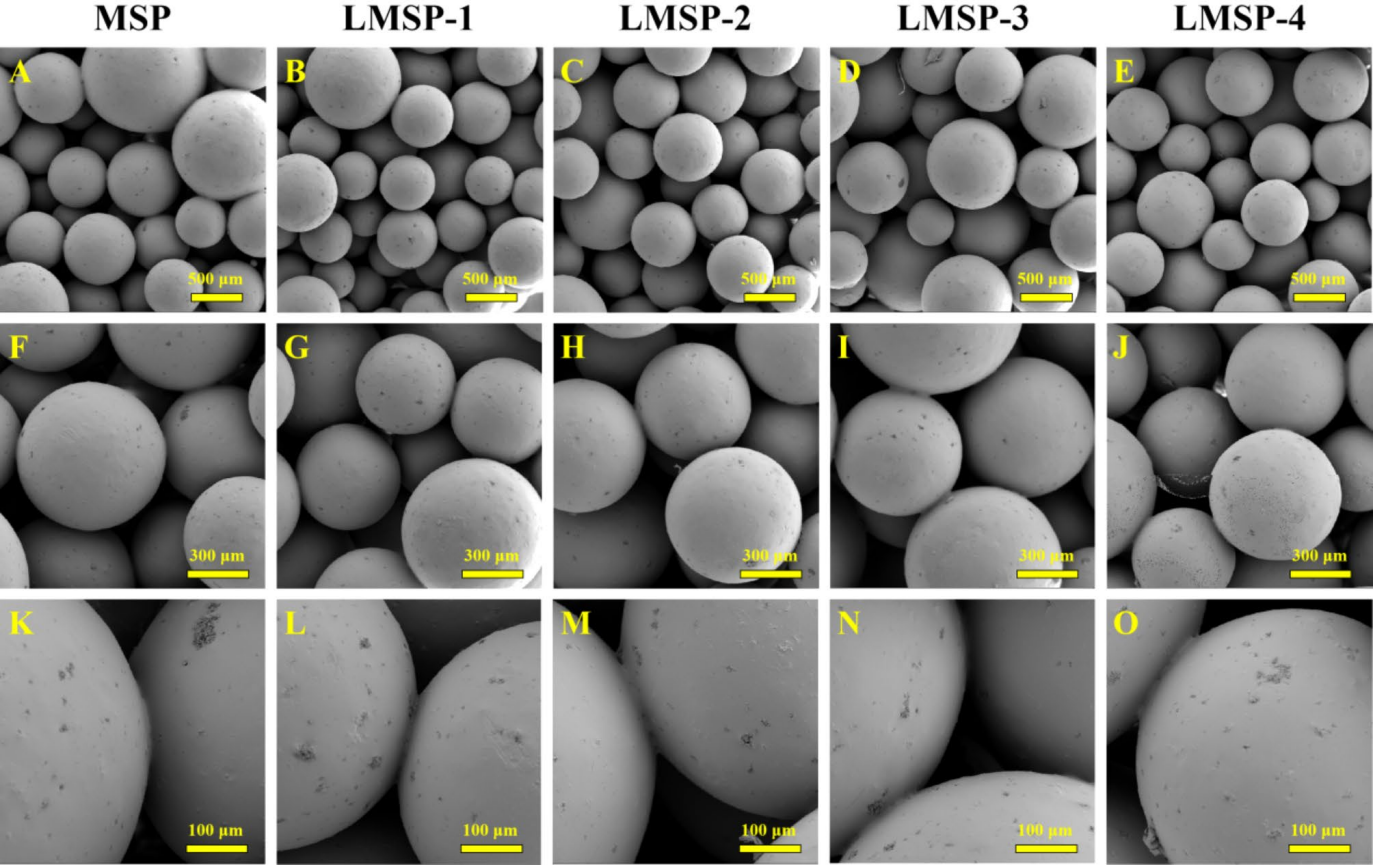
SEM images displaying similar morphology including the effective adhesion between microspheres of MSP and LMSP-1–4 microsphere-based scaffolds prepared in this study.

**Figure 2:**
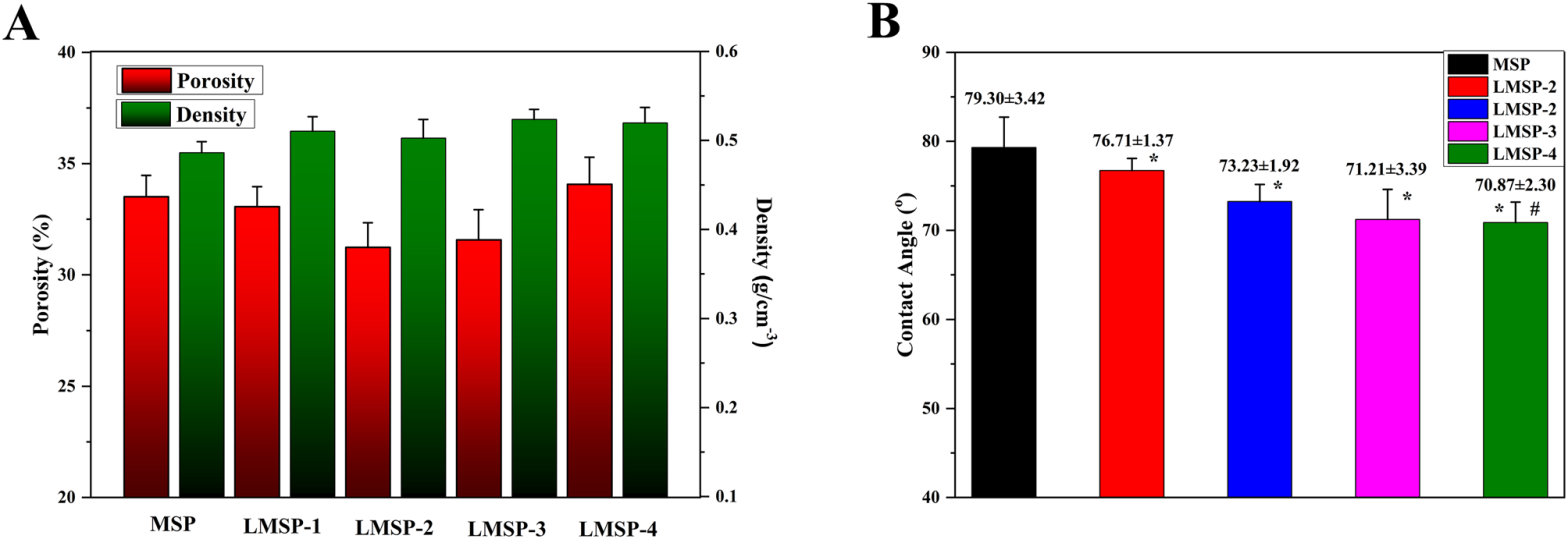
Porosity and density (**A**) and contact angle (**B**) of MSP and LMSP-1–4 scaffolds. (*) and (#) Indicates statistical significance when compared with the MSP and LMSP-1group, respectively (p < 0.05).

Figure 1 shows the morphology of MSP and LMSP-1–4 scaffolds. These scaffolds consist of microspheres bonded to each other, which all have similar 3D porous network structures. And all the microspheres remained spherical. The porosity of these scaffolds is between 30 and 35% (Fig. 2A). The densities of LMSP-1–4 were 0.512 g/cm^3^, 0.503 g/cm^3^, 0.493 g/cm^3^, and 0.486 g/cm^3^ respectively. The compressive strength of MSP and LMSP-1–4 were 4.7 ± 0.42 MPa, 5.3 ± 0.47 MPa, 5.1 ± 0.38 MPa, 4.8 ± 0.61 MPa, and 4.6 ± 0.53 MPa respectively. The content of La in LMS-1–4 increased from 2.16% to 17.20%, and the content of Si in LMS-1–4 ranged from 39 to 62% (Table 2). After LMS-1–4 was added into PLGA to prepare microsphere scaffolds, the Si content decreased to about 5%, and LA could not be detected. This phenomenon indicates that there were few LMS-1–4 particles distributed on the surface of the scaffolds. The content of C from PLGA is about 45%. The slight changes of Si, O, and C contents in MSP and LMSP1–4 groups were related to the spatial distribution of MS or LMSP1–4 in PLGA microspheres.

**Table 2:** Elemental composition (wt%) of MS powder and microsphere-based scaffolds examined by EDS.

MS powders	Si	O	La
MS	39.03	60.97	/
LMS-1	61.97	35.87	2.16
LMS-2	49.29	40.68	10.04
LMS-3	46.87	39.29	13.84
LMS-4	43.17	39.64	17.20

LMSP microsphere-based scaffolds	Si	O	C
MSP	4.96	48.61	46.43
LMSP-1	5.21	49.58	45.20
LMSP-2	5.77	47.10	47.13
LMSP-3	5.10	50.07	44.83
LMSP-4	5.16	46.8	48.04

The contact angles of the LMSP-1–4 composite surface decreased after MS doping La. Compared with the MSP group, with the increase of La doping amount, the surface contact angle decreased significantly, indicating that LMS can improve the surface hydrophilicity of LMSP1–4 composites (Fig. 2B). The contact angle of LMSP-3 was approximately 71.31.

### Release behavior of La^3+^ and change of pH value

**Figure 3:**
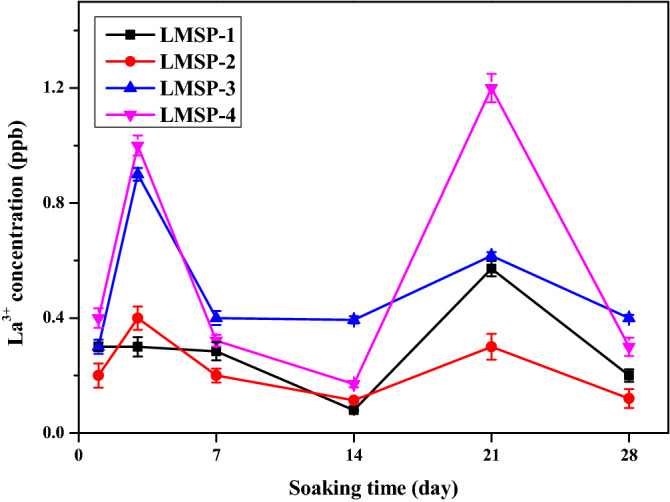
The concentration of La^3+^ released from LMSP-1–4 scaffolds in PBS.

The La^3+^ release behaviors of the LMSP scaffolds were studied by soaking LMSP in PBS at 37 °C for 28 days. At the set time points, the ion concentrations were detected by ICP (Fig. 3). La^3+^ is released rapidly within the first three days. As the soaking time increased, the concentration of La^3+^ began to fluctuate dramatically, except for the group of LMSP-2 and LMSP-4. At all time points, the release amount of La^3+^ in the LMSP-4 group was much higher than that in the LMSP-2 group. What's more, the La^3+^ concentration range of all groups was obtained: 0.079 ± 0.002–1.212 ± 0.05 ppb, which was in the safe concentration range.

**Figure 4:**
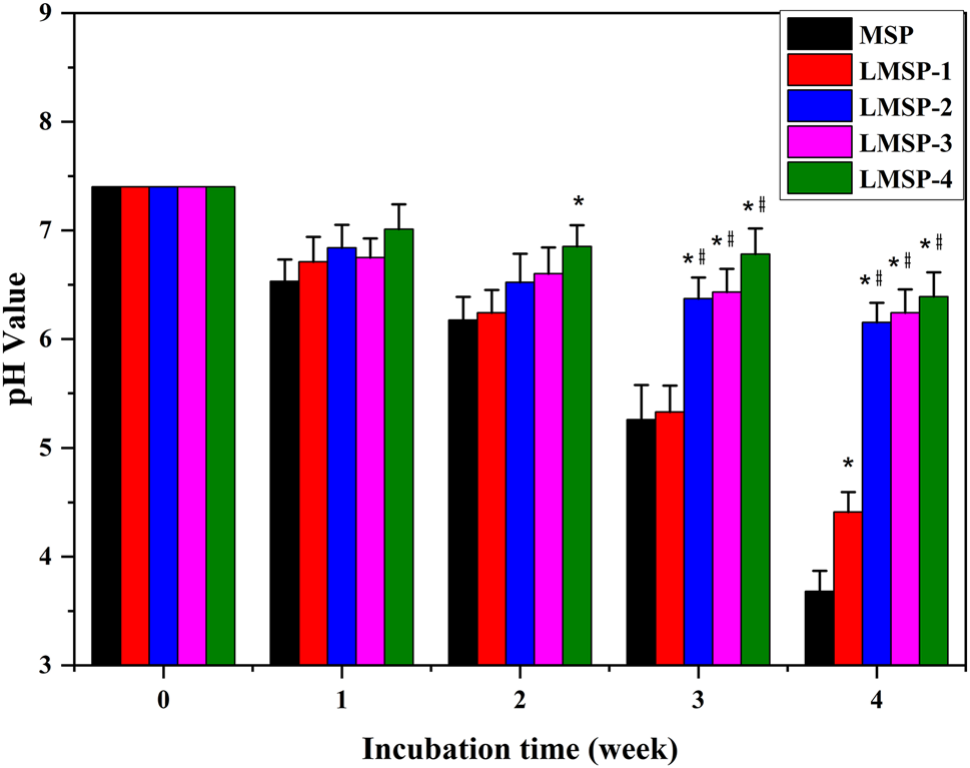
Changes in pH value of PBS during degradation of MSP and LMSP-1–4 scaffolds. (*) and (#) Indicates statistical significance when compared with the MSP and LMSP-1 group, respectively (p < 0.05).

Figure 4 shows the pH change trend of PBS during the degradation of MSP and LMSP microsphere scaffolds. Within 4 weeks, with the increase of La content in the composite scaffolds, the pH value of PBS around the scaffolds increased. At 4 weeks, the pH of LMSP-1–4 groups was significantly higher than the MSP group, and the pH of the MSP group was 3.68 ± 0.19 with that of the LMSP-4 group was 6.39 ± 0.22.

### Cells proliferation

**Figure 5:**
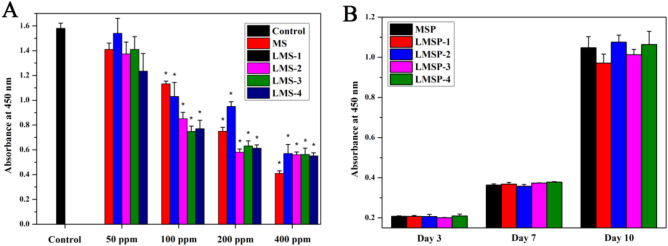
CCK-8 assay for cell proliferation. A: Cells were seeded on MS and LMS-1–4 at concentrations ranging from 50 to 400 ppm for 7 days. (*) Indicates statistical significance when compared with the control group (cells cultured on TCPS without materials) (p < 0.05). B: Cells were seeded on MSP and LMSP-1–4 microsphere-based scaffolds for 3, 7, and 10 days.

Firstly, a CCK-8 assay was used to determine the cytocompatibility of MS and LMS-1–4. According to the detection results of CCK-8, after co-culture with LMS with different amounts of La, the cell viability on day 1 and day 3 showed a similar trend to that on day 7 (data not shown). After 7 days, BMSCs viability in high concentration groups (100, 200, and 400 ppm) decreased significantly compared with the untreated control group (TCPS cultured cells without materials) (Fig. 5A). The inhibition efficiency was enhanced, especially when the La incorporation amounts were increased. The cell viability of the 50 ppm LMS-1–4 groups was close to that of the untreated control group, indicating that the low concentration LMS-1–4 had good cytocompatibility.

**Figure 6:**
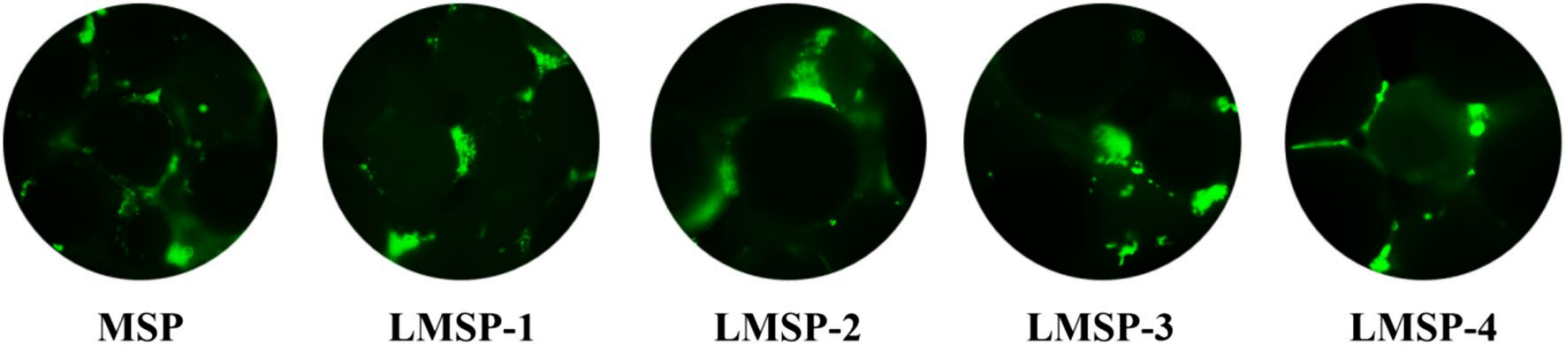
BMSCs cultured on MSP and LMSP-1–4 microsphere-based scaffolds for 24 h were stained with Live/Dead Cell Staining Kit to evaluate the viability of cells implanted on scaffolds.

After 3, 7, and 10 days of culture, the CCK-8 method was used to quantitatively analyze the proliferation of cells on the scaffolds (Fig. 5). The cells seeded on LMSP scaffolds with different La levels continued to grow from day 3 to day 10, indicating that all scaffolds had no cytotoxicity. Compared with the MSP group, the absorbance of the LMSP-1–4 scaffolds is close to that of the control group, and there is no statistical difference. The results showed that the La-doped modification of MS had no adverse effect on the cell proliferation of the scaffold under the experimental conditions. The green fluorescence shown in the live/dead staining images represents living cells, and all scaffolds showed good cell affinity and no cytotoxicity, consistent with the results of the CCK-8 assay (Fig. 6).

### Osteogenesis

**Figure 7:**
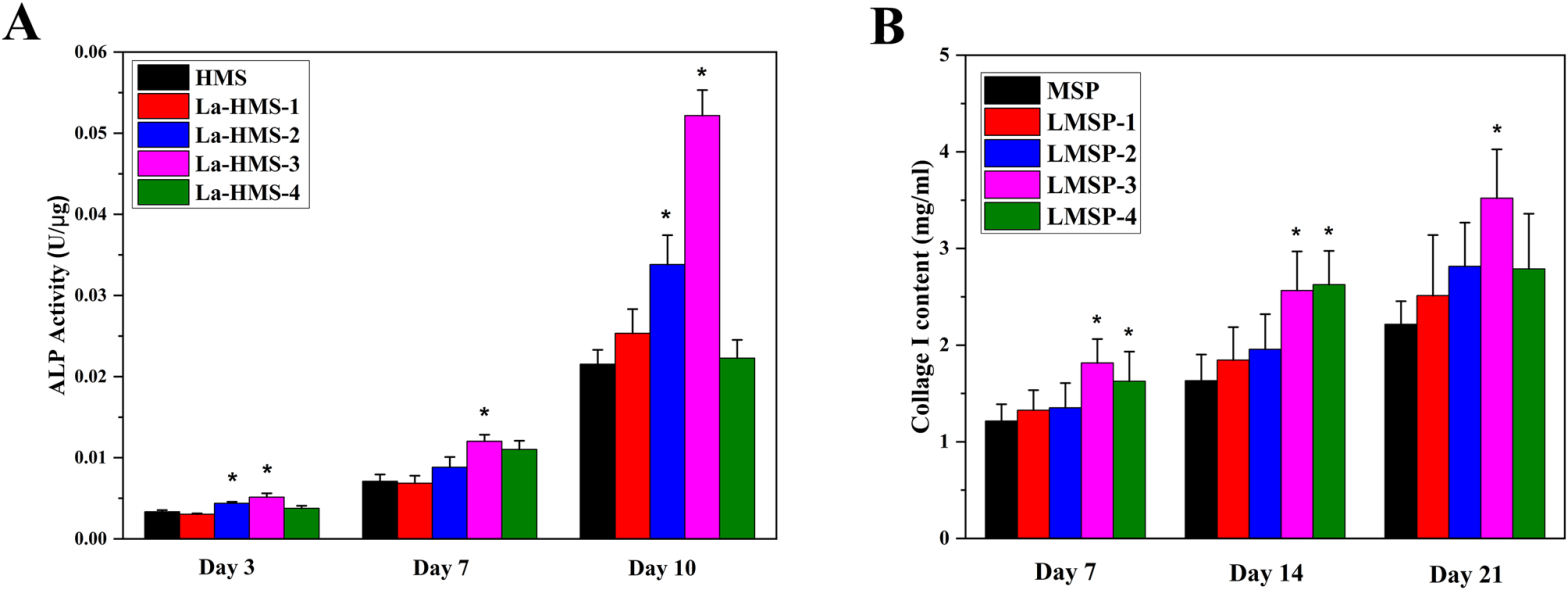
(**A**) ALP activity of BMSCs cultured on scaffolds for 3, 7, and 10 days. (**B**) Collage I content of BMSCs cultured on scaffolds for 7, 14, and 21 days. (*) means the difference was statistically significant (p < 0.05) when made a comparison to the MSP scaffolds.

**Figure 8:**
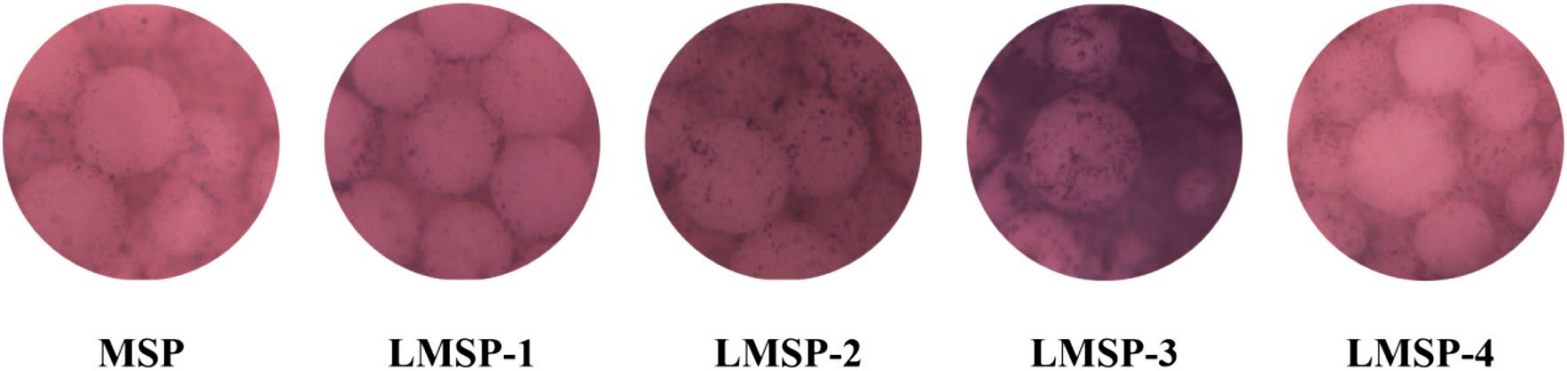
BMSCs cultured on MSP and LMSP-1–4 microsphere-based scaffolds for 10 days were stained with ALP staining Kit to evaluate the ALP secreted from cells.

The ability of microsphere-based scaffolds to induce osteogenic differentiation was determined primarily by the expressions of general marker proteins like ALP and COL-I. The secreted ALP content was analyzed quantitatively after the cells were cultured on the scaffold for 3, 7, and 10 days (Fig. 7A). Cells cultured on LMSP-3 scaffolds showed higher ALP activity at each time point. In the ALP staining results (Fig. 8), the cells showed the same trend after being cultured on the scaffold for 10 days, which was consistent with the quantitative results.

**Figure 9:**
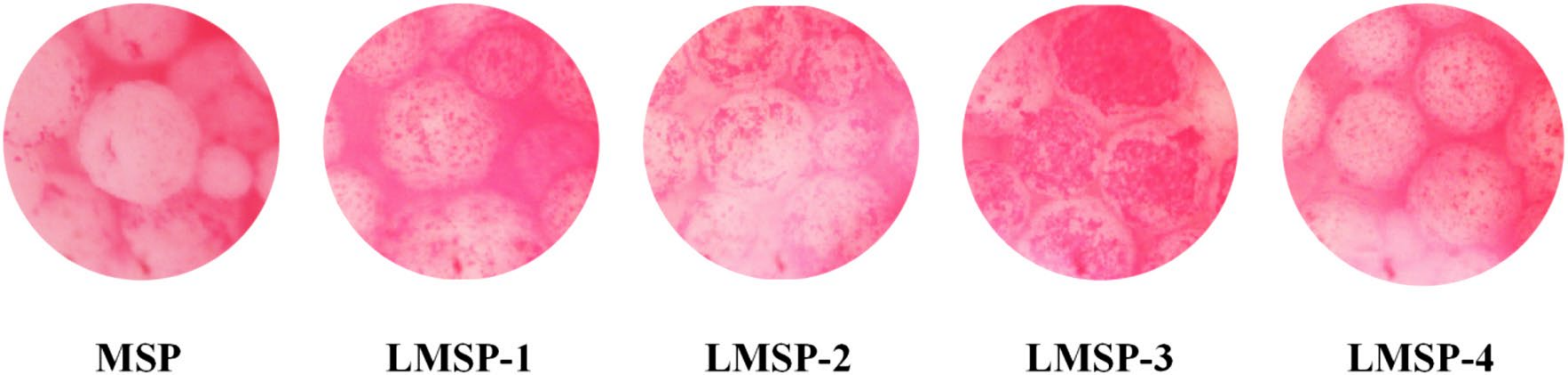
BMSCs cultured on MSP and LMSP-1–4 microsphere-based scaffolds for 21 days were stained with Sirius red staining Kit to evaluate the collagen secreted from cells.

**Figure 10:**
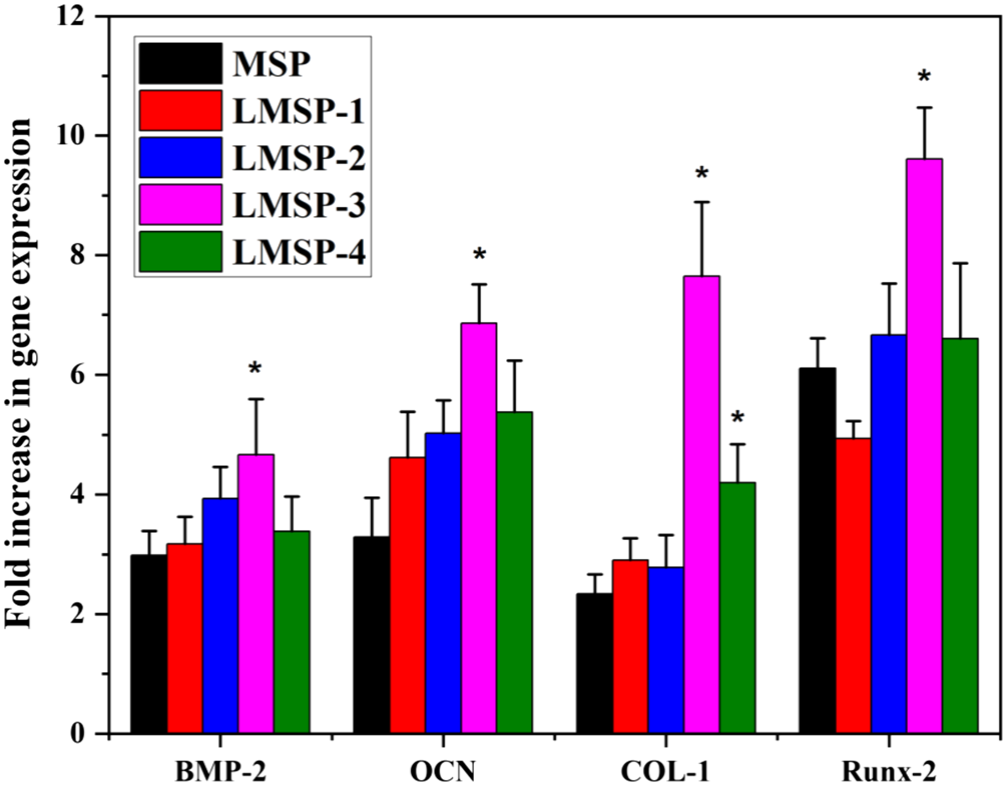
Evaluation of the osteogenic expression of BMSCs seeded and cultured on microsphere-based scaffolds for 21 days via gene expressions of BMP-2, OCN, COL-I, and Runx-2. (*) means the difference was statistically significant (p < 0.05) when made a comparison to the MSP scaffolds.

The secreted COL-1 content was analyzed quantitatively after the cells were cultured on the scaffold for 7, 14, and 21 days (Fig. 7B). The COL-1 content gradually increased along with the inductive culture and displayed a similar ascending trend between groups to that observed for the ALP activity. Overall, BMSCs cultured on LMSP-3 scaffolds showed higher COL-1 content at each time point. As shown in Fig. 9, the collagen secreted by the cells is characterized by Sirius red. The results showed that all components secreted collagen at 21 days. The amount of collagen in the control group (MSP group) was the least, while that in the LMSP-3 group was still the most. It was also consistent with the quantitative results. To further identify the ability to induce osteogenic differentiation, qRT-PCR was applied to detect the expression of osteogenic-related genes (Fig. 10). Results showed a better ability of LMSP-3 microsphere-based scaffolds to induce osteogenic differentiation.

## Discussion

In this study, LMSP scaffolds with 3D porous structures and non-cytotoxicity were constructed for bone tissue regeneration and repair. Compared to MSP scaffolds, LMSP groups not only can release La^3+^ but also can compensate for pH. Moreover, they have better hydrophilicity and osteogenic differentiation ability. The results revealed that the LMSP group could significantly promote the osteogenesis differentiation of the cells. This conclusion can be supported by the biocompatibility, better ALP activity, collagen secretion, and gene expressions of BMP-2, OCN, COL-I, and Runx-2 of BMSCs.

Because microspherical-based scaffolds have excellent initial mechanical properties, they have attracted much attention. To construct LMSP bone tissue engineering scaffolds with good performance, there took the following two preparation steps. First, the PLGA microspheres were prepared via a S/O/W emulsion technique containing LMS particles and La^3+^. Then, prepare microsphere-based scaffolds by low-temperature sintering technique (Fig. 1). The MSP and LMSP-1–4 scaffolds showed 3D interconnected macropores with porosity of 30–35% (Fig. 2A). Compared with 3D printed scaffolds, the porosity of microsphere sintered scaffolds is much smaller. However, in this study, microsphere sintered scaffolds had visible micropores (more than 100 μm in size), and these sizes allowed the ingrowth, proliferation, and differentiation of bone-associated cells^28,36,37^.

When combined with PLGA, LMS enhanced the osteogenic differentiation ability of BMSCs without affecting the cellular activity of cells. From the results of the CCK-8 testing, the LMSP scaffolds showed no cytotoxicity (Fig. 5B). The porous structure of the scaffolds facilitates the cells to enter into and promotes communication between cells. BMSCs enter the scaffolds through channels and adhere closely to the surface of the scaffold (Fig. 6). This result is consistent with the findings of others that cell viability was not affected after cells were grown on scaffolds doped with appropriate amounts of La-doped material^18^. However, LMS-1–4 at high concentrations (100, 200, and 400 ppm) have cytotoxicity (Fig. 5A). Only 50 ppm of LMS-1–4 had good cytocompatibility. According to reports, apoptosis may occur when La^3+^ concentration exceeds 10^–5^ M^15,38^. It may be because when the concentration of LMS-1–4 is higher, they also release a higher concentration of La^3+^, thus reducing the survival rate of BMSCs. In this study, at all the time points, the concentration of La^3+^ ions released from the LMSP scaffold was only about 0.1–1.2 ppb (Fig. 3), which was within the safe concentration range^17,39^.

PLGA-based microspheres have been known as drug carriers for decades. The release of drugs from microspheres depends on drugs diffusion through the polymer matrix and the degradation of the polymer^40^. However, compared with drugs, the release kinetics of La^3+^ from microspheres is mainly controlled by La-doped material because the diameter of La^3+^ is tiny (only about 0.21 nm). The degradation rate of Si-based materials is closely related to their chemical composition^15^. In this study, the residual template in the preparation of LMS was removed by calcination, and the degradation rate of LMS was slow due to crosslinking caused by high temperature^21^. We adjusted the doping amount of La in LMS and incorporated LMS-1–4 into PLGA to obtain an adjustable release behavior of La^3+^. When PLGA was mixed with a relatively small amount of LMS-1–4 to prepare microsphere-based scaffolds, only about 5% Si and La could not be detected on the surface of scaffolds due to the small amount of LMS-1–4 distributed on the surface of scaffolds (Fig. 1, Table 2). When LMS is encapsulated by PLGA, the release of La^3+^ from LMS will be inhibited. ICP results showed that the concentration of La^3+^ fluctuated during the whole soaking process (Fig. 3). During the first 3 days of soaking, the concentration of La^3+^ in the solution increased with the continuous degradation of LMS. When more La sources are added, more La replaced Si, forming more Si–O–La bonds, resulting in higher La content and lower Si content in LMS (Table 2). At the same time, the difference between the diameters of La and Si atoms will lead to more defects in the mesoporous skeleton. This further accelerated the degradation rate of LMS and La^3+^ release, in agreement with other studies^16,19^. Compared with LMS-1–3, the addition of more La sources in the preparation process of LMS-4 leads to more lattice defects. This may be the reason for the larger amount of La^3+^ release from LMSP-4 than from LMSP-1–3 and the larger fluctuations in the release profile throughout the release process. P in PBS is deposited on LMSP to form a phosphate layer, which will gradually hinder the diffusion of La^3+^, so the concentration of La^3+^ in PBS fluctuates^41,42^. Meanwhile, a quarter of PBS was replaced at each time point, which also resulted in a decrease in the concentration of La^3+^.

In essence, multiple factors at the gene and protein levels governed BMSCs osteogenic differentiation. La^3+^ plays an important role in improving the ability to induce osteogenic differentiation of scaffolds. As an early marker of bone formation, ALP is mainly expressed on the cell surface or in stromal vesicles and has a significant regulatory effect on phosphate supplementation during bone mineralization^39^. As an early-stage marker that is observed in the preparation stage of osteogenic differentiation, ALP activity was increased significantly in the LMSP-2–3 group especially in the LMSP-3 group (Figs. 7A, 8). The initial stage of collagen biosynthesis plays an important role in the development of mature bone tissue^43,44^. In this study, the osteoblasts in the LMSP-3 group also showed significantly higher collagen gene and protein secretion (Figs. 7B, 9, 10). BMP-2, OCN, and Runx-2 also take key roles in bone formation. Compared with other groups, LMSP-3 microsphere-based scaffolds showed the higher BMP-2, OCN, and Runx-2 gene expression (Fig. 10). This finding showed that compared with MSP scaffolds, LMSP-1–4 scaffolds especially LMSP-3 scaffolds, have higher osteogenic induction. LMSP-3 concentration in the right conditions could steadily release suitably does of La^3+^, which could support proliferation and promote the osteogenesis differentiation of BMSCs effectively. Previous studies have shown that La^3+^ at concentrations of 0.1–1.0 ppb had promoted the osteogenic differentiation of BMSCs and MC3T3-E1 at a range of concentrations. So did-containing materials such as La-substituted layered double hydroxide nanohybrid scaffolds^15,18,41,45^.

PLGA was approved by the FDA for use in humans several years ago and is ideal for preparing tissue engineering materials and drug carrier materials. It has many advantages, including good biocompatibility, adjustable degradation rate, good mechanical properties, and is easy to shape. However, PLGA degrades into water and CO_2_ in vivo, and access CO_2_ leads to increased acidity in the vicinity, which is prone to a local aseptic inflammatory reaction and clinical treatment failure. It is a major common obstacle in developing PLGA-based materials for tissue engineering especially bone tissue engineering. As we all know, the autocatalysis of PLGA may accelerate its degradation^46^. In this study, during the degradation of MSP and LMSP, the acidic degradation products produced by PLGA in the scaffolds were gradually dispersed into the medium, which resulted in the decrease of pH value (Fig. 4). For the LMSP-2–4 group, the pH remained above 6 throughout 4 weeks, which was significantly higher than the MSP and LMSP-1 group. According to our previous studies, LMS is more alkaline than MS. With the increase of the doping amount of La, LMS has more strong alkalinity^34^. It is mainly because La-MS could neutralize acid products. And the material has a more pH compensation effect. Therefore, LMS can balance the acidity caused by PLGA degradation and may effectively alleviate the immune response caused by the acidic environment in the clinic. After BMSCs were inoculated onto scaffolds and cultured with OGM, they could express osteogenic markers. Compared with MSP scaffolds, the ability of osteogenic differentiation of BMSC is stronger in the La-doped MSP scaffolds, which laid a foundation for the development of La-containing 3D porous microsphere-based scaffolds for bone repair.

## Conclusion

Given the vital regulatory role of La in osteogenesis, it is of great significance and value to study the La^3+^ release kinetics of biomaterials targeting bone regeneration to achieve stable and controlled release of La^3+^. In the current attempt, the steady release behavior of La^3+^ has been controlled successfully by incorporating LMS with different amounts of doped La into bioresorbable PLGA microsphere-based scaffolds. These LMSP microsphere-based scaffolds provided feasibility to regulate the ALP and COL-1 secretion and osteogenic differentiation at a certain concentration of La^3+^. Based on this study, these LMSP microsphere-based scaffolds with La^3+^ stable controlled release kinetics can be applied for further research to deepen the understanding of the role of La^3+^ in promoting bone regeneration or other possible biological effects, such as bone immune regulation.
